# Nurses' Visual Attention During Initial Observation of Patients With Suspected Pneumonia: An Observational Pupillometry Study

**DOI:** 10.1111/jjns.70076

**Published:** 2026-09-03

**Authors:** Shoya Kamijo, Shinji Matsutani, Eijun Nakayama, Naoya Ohtani

**Affiliations:** ^1^ School of Nursing Kitasato University Sagamihara Kanagawa Japan; ^2^ Tokyo Medical University School of Nursing Tokyo Japan; ^3^ Kitasato University Hospital Sagamihara Kanagawa Japan

**Keywords:** attention, clinical competence, clinical deterioration, eye movements, eye‐tracking technology, pupil

## Abstract

**Aim:**

Observation requires selective attention to visual information acquired through gaze. Measuring pupil diameter, an index of attention, we examined skilled and novice nurses during the initial assessment of a patient urgently admitted with suspected community‐acquired pneumonia.

**Methods:**

This observational study was conducted in a simulated environment. Participants comprised skilled nurses with advanced practice training and novice nurses. During a 120‐s initial assessment of a mock patient with suspected community‐acquired pneumonia, participants wore eye‐tracking equipment. Pupil diameter and gaze transitions were recorded. Changes in pupil diameter were determined by subtracting the baseline value from the measured pupil diameter and compared while participants focused on specific regions such as the face.

**Results:**

A total of 16 skilled nurses and 17 novice nurses participated. Novice nurses showed greater negative changes in pupil diameter than skilled nurses when focusing on the facial and chest areas. Mixed‐effects modeling showed that, in the later phase of the task, novice nurses exhibited smaller changes in pupil diameter, whereas skilled nurses exhibited slightly larger changes.

**Conclusions:**

Compared with novice nurses, skilled nurses directed their attention more intensively to the face and chest during the initial assessment of a patient with suspected community‐acquired pneumonia and impaired respiratory status. They also showed slightly larger changes in pupil diameter in the later phase of the task than novice nurses, suggesting the engagement of selective attention. These findings provide a basis for developing educational materials to train nurses to respond effectively to sudden changes in a patient's condition.

## 1 Introduction

Observation is a core nursing skill across various clinical settings (Alastalo et al. 2017; Hamilton and Manias 2007). On hospital wards, nurses detect signs of clinical deterioration through observation (Liaw et al. 2011). However, these critical observations remain largely tacit, making them difficult to teach effectively to novice nurses. Recently, eye‐tracking has been used to characterize nursing observation techniques and make these skills visible. Studies of nurses' actual eye movements have shown that those who were proficient at recognizing errors made frequent gaze transitions, including across patient charts (Marquard et al. 2011; Marquard et al. 2013); moreover, experienced nurses spent more time gazing at critical areas such as the surgical incision site during cesarean section assistance (Koh et al. 2011). In eye‐tracking research on nursing observation, the outcomes used to characterize gaze behavior have typically been gaze duration (Grundgeiger et al. 2010; Henneman et al. 2017; He et al. 2014; Hofmaenner et al. 2021; Koh et al. 2011) or the number of fixations (He et al. 2014; Hofmaenner et al. 2021; Marquard et al. 2011). However, healthcare professionals may fail to recognize what they are looking at when attention is lacking (Drew et al. 2013; Jones and Johnstone 2017). Therefore, characterizing observation skills requires the assessment not only of gaze patterns but also of attentional states. According to a scoping review of both English and Japanese literature, a key challenge in elucidating nurses' observation skills lies in objectively identifying the direction of attention (Kamijo et al. 2023). Some studies have used pupil diameter as an indicator of cognitive load rather than explicitly defining attention. Cabrera‐Mino et al. (2019), examining a 10‐min observation of patients with heart failure, suggested that psychological stress may influence cognitive load, possibly because of the prolonged observation period. However, emergency situations involving patients with deteriorating conditions require nurses to make rapid, momentary assessments. In these situations, gazing at the patient is not sufficient; nurses must actively direct attention (Jones and Johnstone 2017) to rapidly comprehend the patient's condition. Therefore, clarifying the attentional demands of nursing observation is essential for improving patient safety.

Attention underlies the selection of sensory information (Atkinson and Shiffrin 1968; Waugh and Norman 1965) and is generally categorized into two types: (1) goal‐directed, top‐down attention driven by knowledge and expectations; and (2) stimulus‐driven, bottom‐up attention driven by the novelty or salience of stimuli. These forms of attention are thought to be mediated by distinct regions of the cerebral cortex (Corbetta and Shulman 2002; Xia et al. 2024). Beyond the cortex, the pontine locus coeruleus is also known to contribute to attentional processing (Bari et al. 2020; Ghosh and Maunsell 2024). Recent studies have shown that these brain regions exhibit activity associated with pupil dilation (Alnæs et al. 2014; Bradley et al. 2008; Joshi et al. 2016; Murphy et al. 2014; Privitera et al. 2020); pupil diameter is now widely used as an indicator of attention (Joshi and Gold 2020; Strauch et al. 2022). Nurses' observational skills can be visualized and translated into explicit knowledge by recording their gaze behavior and pupil diameter changes during observation. These findings could also inform the design and color of visual targets that require focused attention.

Virtual reality (VR)‐based educational materials are increasingly used in nursing education. A systematic review showed that VR use in nursing education improves practical skills more effectively than traditional methods (Liu et al. 2023). As VR enables immersive, controlled environments, it is suitable for training in rare but critical situations. Objective data on nurses' observation skills could therefore inform the development of VR‐based educational materials.

In this study, we recorded the pupil diameter—an index of attention—of nurses who had completed advanced practice training (hereafter referred to as “skilled nurses”) and compared it with that of novice nurses during the observation of critically ill patients. We hypothesized that skilled nurses would be more likely to direct their attention to critical areas during patient assessment. Furthermore, we anticipated that skilled nurses would deploy selective attention more readily than novice nurses during the initial observation of patients admitted in emergency situations. Therefore, this study aimed to identify differences in attention patterns between the two groups and to clarify the distinctive attentional characteristics of skilled nurses.

### 1.1 Theoretical Framework

**FIGURE 1:**
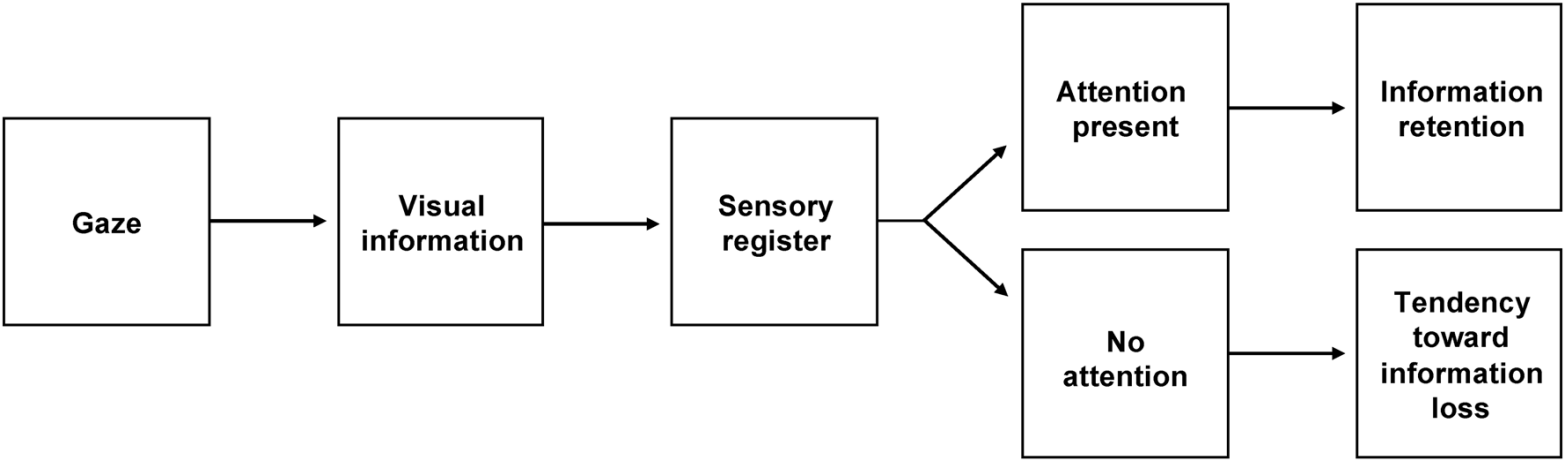
Conceptual framework of the study. Gaze is defined as projecting a target onto the retinal fovea, and the information obtained through gaze is sent as visual information to the sensory register for temporary storage. Information that receives attention is retained, whereas unattended information tends to fade. The figure schematically illustrates, using directional arrows, the process of selective attention to visual information obtained through gaze.

In this study, nurses were asked to observe a patient according to a standardized scenario in a simulated environment. A theoretical framework was developed on the premise that attention is related to the selection of information (Atkinson and Shiffrin 1968; Waugh and Norman 1965) and that gaze and attention are closely linked (Corbetta et al. 1998) (Figure 1).

According to the model proposed by Atkinson and Shiffrin (1968), visual information is acquired by gazing at the target and transferred to the sensory register. Important information within this register must be selected through attention because sensory input rapidly decays. Selected information is transferred to memory systems such as short‐term memory, whereas unselected information tends to disappear. Because attention is considered to be linked to gaze control mechanisms, the period from gaze fixation to the selection of information through attention was defined as “observation,” and both gaze and pupil diameter data were collected. In this study, attention was operationally defined as a state of pupil dilation.

## 2 Methods

### 2.1 Study Design

This observational study was conducted in a simulated environment to record nurses' gaze toward a patient and the surrounding environment, as well as changes in pupil diameter during the initial assessment of a patient urgently admitted with suspected community‐acquired pneumonia.

### 2.2 Scenario Development for the Experiment

We first developed an experimental scenario and assessed its clinical relevance. Community‐acquired pneumonia is a major cause of infectious disease‐related death worldwide (Tejada et al. 2018); in Japan, more than 90% of deaths among adults occur in individuals aged 65 years or older (Miyazaki et al. 2023). As community‐acquired pneumonia often presents with respiratory symptoms, patients with this condition require careful respiratory monitoring. Respiratory indicators are important signs of clinical deterioration (Lynn and Curry 2011; Schein et al. 1990). Because this disease is common in older adults and can lead to respiratory deterioration, understanding how nurses allocate attention to such patients is clinically important. We therefore selected this condition for the scenario. Based on this context, we developed a case depicting a nurse conducting an initial assessment of an older woman with impaired consciousness. The woman was transported to the emergency department with suspected community‐acquired pneumonia, underwent several tests as an outpatient, and was then urgently admitted to a general ward. This scenario was designed so that the patient's condition deteriorated during the observation period; however, the underlying cause of the deterioration was intentionally left unspecified because patients requiring emergency admission may still be undergoing diagnostic evaluation and may not yet have a definitive diagnosis. This design also allowed participants to consider multiple possible clinical scenarios when assessing the patient's condition. This scenario allowed us to evaluate whether nurses directed their attention to the patient's condition to detect early clinical deterioration.

#### 2.2.1 Scenario Evaluators

As tasks related to clinical practice require simulation of real‐world settings to ensure validity (Cioffi 2001), the developed scenario was divided into six components: (1) pre‐admission setting, (2) vital signs and physical findings in the ER, (3) test results from the ER, (4) patient condition upon arrival at the ward, (5) deterioration in the patient's condition, and (6) key areas for visual attention. These six items were evaluated for clinical relevance in the context of a deteriorating case of suspected pneumonia by nurses certified in emergency nursing and respiratory nursing by the Japan Nursing Association. Additional evaluators included nurses who had served on a hospital Respiratory Support Team and nurses involved in in‐hospital simulation‐based education. To include both nursing and medical perspectives, a board‐certified member of the Japanese Respiratory Society was asked to evaluate the scenario. In total, five experts were recruited to evaluate the scenario.

#### 2.2.2 Scenario Content Validity Assessment

Each evaluator received a form listing the six scenario items and was asked to rate each item's relevance to clinical practice on a four‐point scale (1 = Not relevant to 4 = Highly relevant).

Responses of 4 (Highly relevant) or 3 (Quite relevant) were assigned a score of 1 (valid), whereas 2 (Somewhat relevant) or 1 (Not relevant) were assigned a score of 0 (not valid). Based on these scores, an item‐level content validity index (I‐CVI) was calculated for each item by dividing the number of evaluators who judged the item as valid by the total number of evaluators. According to Lynn (1986), when there are five or fewer evaluators, agreement on all items is recommended to ensure validity. This criterion was adopted in the present study for the evaluators. The I‐CVI was 1.00 for all items. Therefore, the scenario was determined to be clinically relevant (Table 1).

**TABLE 1:** Evaluation of the items in the experimental scenarios.

Written scenario items	Rater					I‐CVI
	ID.1	ID.2	ID.3	ID.4	ID.5	
1. Pre‐hospitalization scenario setting	3	4	4	4	4	1.00
2. Vital signs and physical findings in the outpatient setting	4	4	3	4	4	1.00
3. Diagnostic test results from the outpatient visit	4	4	4	4	4	1.00
4. Patient condition upon arrival to the ward	4	4	4	4	4	1.00
5. Clinical deterioration scenario	4	4	3	4	4	1.00
6. Key gaze areas of interest	4	4	3	4	4	1.00

*Note:* Ratings of 4 (highly relevant) and 3 (quite relevant) were recoded as 1 (valid), whereas ratings of 2 (somewhat relevant) and 1 (not relevant) were recoded as 0 (not valid). ID.1 is a nurse certified as a Certified Nurse in Emergency Nursing by the Japanese Nursing Association. ID.2 is a nurse certified as a Certified Nurse in Respiratory Nursing by the Japanese Nursing Association. ID.3 is a nurse with prior experience as a member of the in‐hospital Respiratory Support Team. ID.4 is a nurse with experience in conducting in‐hospital simulation‐based nursing education. ID.5 is a physician board‐certified in respiratory medicine by the Japanese Respiratory Society.

This validated scenario was used in the present study (Table 2).

**TABLE 2:** Content of the scenario used in the experiment.

Written simulation scenario
*1. Pre‐hospitalization scenario setting* An 80‐year‐old woman lives in a single‐family home with her 80‐year‐old husband. Her medical history includes hypertension and dyslipidemia. She had been taking antihypertensive and lipid‐lowering medications prescribed by her local physician. Approximately one week ago, her food intake began to decline. Since the previous day, she had become increasingly lethargic and developed a fever of 38°C. Concerned about her condition, her husband called emergency medical services. Upon arrival, the emergency team found her oxygen saturation (SpO_2_) to be 88%. She was transported to our hospital by ambulance with supplemental oxygen. She was admitted for further evaluation and treatment.
*2. Vital signs and physical findings in the emergency outpatient setting* The patient's vital signs were as follows: blood pressure 132/62 mmHg, heart rate 105 bpm with sinus rhythm, respiratory rate 22 breaths per minute, and body temperature 38.8°C. While receiving oxygen via nasal cannula at 2 L/min, her oxygen saturation (SpO_2_) was approximately 94%. She presented with a cough. On auscultation, coarse crackles were heard in the mid‐to‐lower right posterior lung fields. Her level of consciousness was assessed as JCS II‐10. She was able to vocalize unintelligibly (“Uhh”) and showed signs of distress on her face.
*3. Diagnostic test results from the emergency outpatient visit* Laboratory findings revealed a white blood cell count of 12.0 × 10^3^/μL, C‐reactive protein (CRP) of 16.0 mg/dL, blood urea nitrogen (BUN) of 18.0 mg/dL, and creatinine of 0.7 mg/dL. Arterial blood gas analysis showed pH 7.44, PaO_2_ (70.0 Torr), PaCO_2_ (35.0 Torr), HCO_3_^−^ (24.0 mEq/L), and base excess (BE) (1.0 mEq/L). Results from various cultures were still pending. A 12‐lead electrocardiogram (ECG) showed sinus rhythm, and a chest X‐ray revealed an infiltrative shadow in the right lower lung field.
*4. Patient condition upon arrival to the ward* After undergoing examinations and initial treatment in the emergency outpatient department, the patient was transported by stretcher to a private room in the hospital ward for admission. Upon arrival, she was fully assisted by nursing staff to transfer herself to the bed and was placed in the supine position. A peripheral intravenous line was inserted into her right forearm, through which 500 mL of normal saline was being administered at a rate of 40 mL/h. She was receiving oxygen via nasal cannula at 2 L/min. Electrodes and probes were attached to continuously monitor the heart rate, lead II ECG waveform, SpO_2_, and respiratory rate using a bedside monitor.
*5. Clinical deterioration scenario* Sixty seconds after the initial assessment upon arrival at the ward, the respiratory rate increased to 32 breaths per minute, SpO_2_ decreased to 88%, heart rate rose to 110 beats per minute, and blood pressure changed to 142/70 mmHg. The JCS score remained at II‐10, and body temperature stayed unchanged at 38.8°C.
*6. Key gaze areas of interest* Facial area, chest area, bedside monitor, oxygen flowmeter (including suction and air piping), and patient information sheet

### 2.3 Participants

The participants were recruited between September 29, 2023, and March 22, 2024, at a university hospital in Japan using posters and snowball sampling. Recruitment was not limited to specific wards; instead, the participants were recruited across a wide range of departments. Eligible participants comprised two groups: skilled nurses who had completed the Specified Medical Acts Training Program and novice nurses with at least one but fewer than 2 years of clinical experience. Japan has a Specified Medical Acts Training Program, which allows nurses to perform sophisticated medical procedures under physician supervision (Ministry of Health, Labour and Welfare, n.d.). Specified medical acts include adjusting settings for invasive positive‐pressure ventilation or modifying insulin dosages and are categorized into 21 areas. To perform these acts, nurses must complete a set of mandatory courses that enhance practical understanding, critical thinking, judgment, and advanced knowledge and techniques. These courses cover topics related to diseases, pathophysiology, physical assessment, and clinical reasoning and are designed to develop nurses' decision‐making and thinking skills. Therefore, nurses who have completed these core courses are considered competent to observe patients with serious conditions and identify abnormalities and are defined as skilled nurses for the purpose of the present study.

**FIGURE 2:**
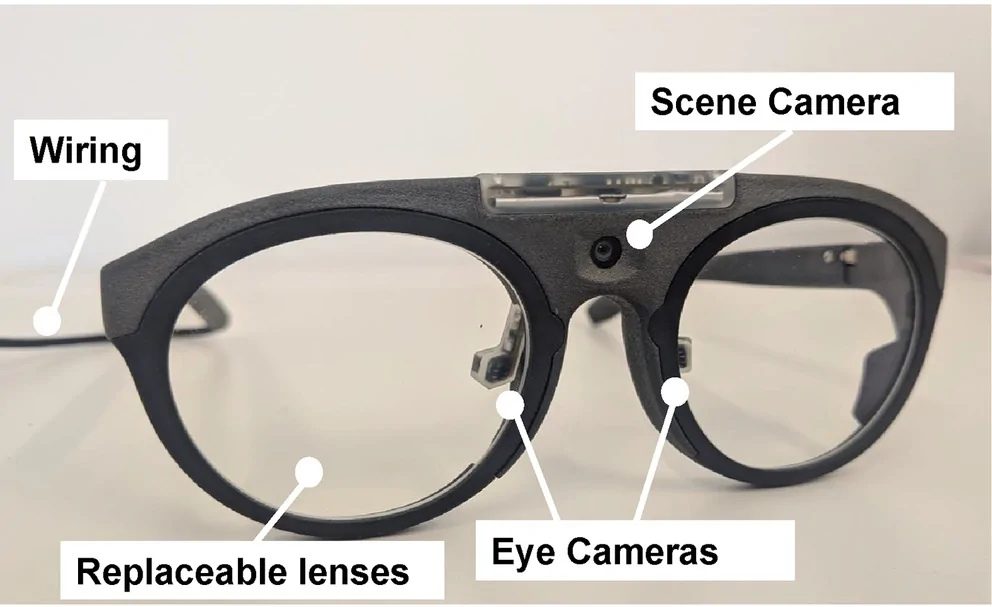
The Neon device. The scene camera records at 30 Hz. The infrared eye cameras, located on both sides, record eye movements and pupil size at 200 Hz. The wiring visible on the left extends from the end of the temple and is connected to an Android smartphone. The left and right lenses can be replaced with prescription lenses as needed.

The eye‐tracking device used in this experiment was the Neon (Pupil Labs GmbH, Berlin, Germany), which allows lenses to be replaced with prescription lenses ranging from −3 to +3 (Figure 2). Because the device is compatible with contact lenses, LASIK, and implantable contact lenses, no issues with measurement were expected. However, participants who typically wore glasses and whose prescription was outside the −3 to +3 range—who would be unable to see adequately without them—were excluded because of the device's glasses‐like design. Additionally, individuals who were unable to provide written informed consent were excluded.

The sample size was calculated using the power analysis software G*Power (Version 3.1.9.7; Faul et al. 2007). Using a two‐tailed *t*‐test with an effect size of *d* = 1.17, *α* = 0.05, and power = 0.80, we determined that 13 participants were required in each group. The effect size was calculated based on a similar study (Okane et al. 2022). To account for possible dropouts, the target recruitment number was increased by three per group, yielding a target sample of 16 skilled nurses and 16 novice nurses.

### 2.4 Experimental Setting

**FIGURE 3:**
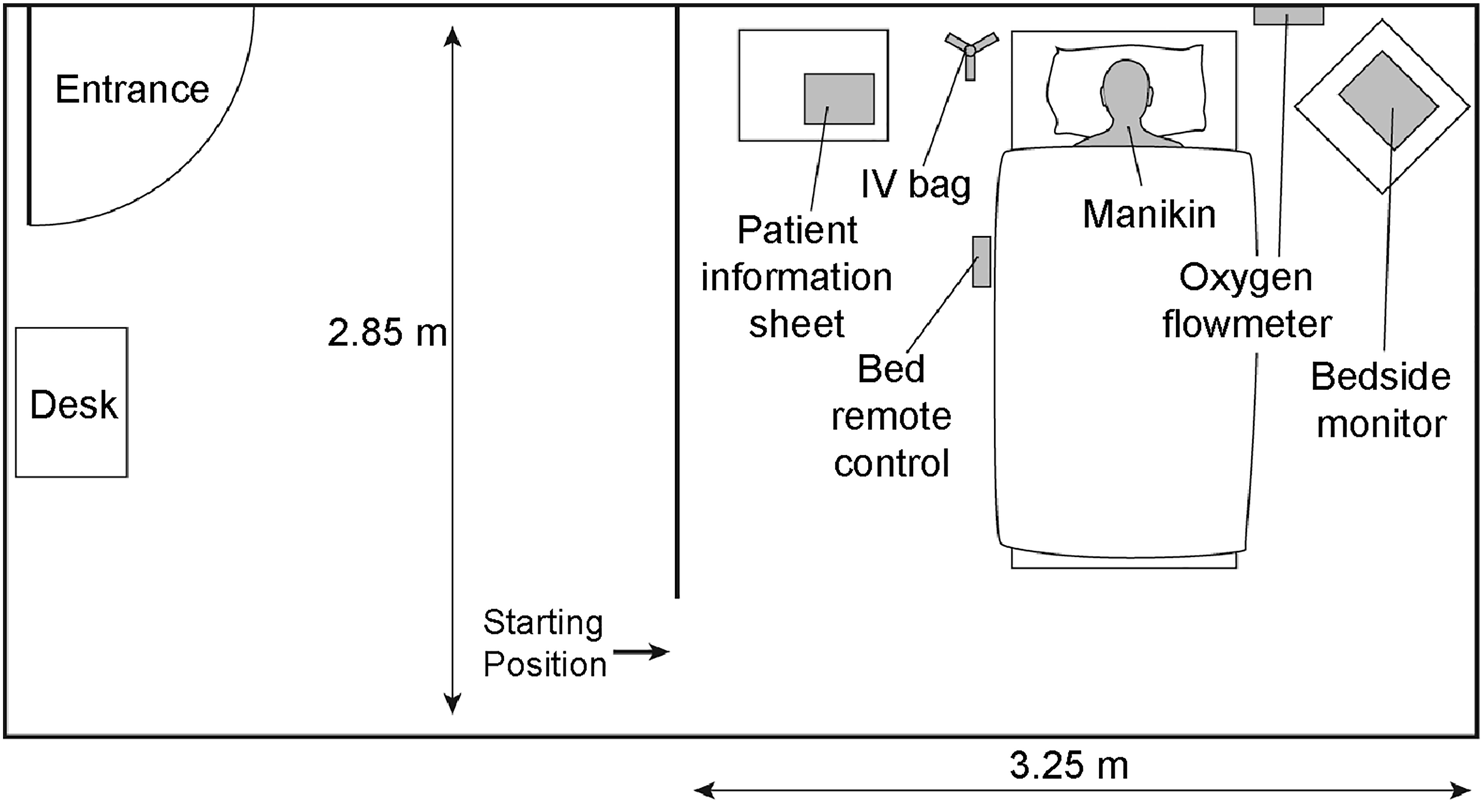
Laboratory layout. The oxygen flowmeter includes suction and air piping. A penlight, stethoscope, and thermometer were placed adjacent to the patient information sheet. The blood pressure cuff was positioned below the bedside monitor, with a tablet placed on its screen area to display heart rate, SpO_2_, respiratory rate, and ECG waveform through the TruMonitor app. The nurse commenced observation from the position indicated as “Starting Position” and was able to move around the left and right sides of the bed as well as the foot of the bed to gaze at the target objects. The manikin was positioned at the center of the observation space. IV = intravenous.

The experiment was conducted in a university laboratory (Figure 3). As natural light from windows can vary depending on the weather and time of day and affect pupil diameter measurements, the windows were covered with boards, mirrors were removed, and only fluorescent lighting was used.

### 2.5 Experimental Procedure

First, the experiment was explained to the participants at a desk near the laboratory entrance. Subsequently, they completed a demographic questionnaire. They were then shown the patient information for 60 s to understand the context of the observation. The patient information sheet was placed near the bed during the task for easy access. The participants were not informed beforehand that the patient's condition would deteriorate 60 s after observation began. A nonfunctional manikin played the role of the patient. To simulate the respiratory rate, a prerecorded audio file was played through a SoundLink Mini Bluetooth speaker II (Bose Corporation, Framingham, Massachusetts) placed under the bed. A Galaxy Table S8+ (Samsung Electronics Co. Ltd., Seoul, South Korea) running the TruMonitor application (Laerdal Medical AS, Stavanger, Norway) displayed the respiratory rate, SpO_2_, heart rate, and ECG waveform. The sound and TruMonitor application were controlled using a second Galaxy Table S8+.

The simulation task consisted of a 120‐s observation period. The participants wore the Neon device and were escorted to a simulated hospital room divided by partitions. If participants spoke to the manikin during observation, the researcher responded with groaning sounds in place of the patient's voice. If the participants measured vital signs before the patient's condition changed, the values listed in the patient information sheet corresponding to the ER data were provided. At the 60‐s mark, the patient's condition was changed on the TruMonitor app to a respiratory rate of 32 breaths/min, SpO_2_ of 88%, and heart rate of 110 bpm. The audio data were also switched to reflect the new respiratory rate of 32 breaths/min. If participants measured vital signs after this change, the updated values reflecting the patient's change in condition were provided. Physical findings from the patient information sheet were provided for any other observations. For items not listed, the researcher stated that there were no abnormal findings. The end of the task was signaled at the 120‐s mark.

### 2.6 Measurement Methods

First, the participants were asked to complete a demographic questionnaire indicating age, sex, and whether they used vision correction, including the type of correction. Using the Neon device, binocular gaze data and pupil diameter were recorded at 200 Hz, and scene‐camera video was recorded at 30 Hz. The data were saved on a wired Android smartphone (OnePlus 10 Pro; OnePlus Technology Shenzhen Co. Ltd., Shenzhen, China) and then uploaded to the analysis platform Pupil Cloud (Pupil Labs GmbH).

### 2.7 Data Analysis

#### 2.7.1 Demographic Data

Nominal and interval‐scale variables were categorized and compared between the skilled and novice nurse groups.

#### 2.7.2 Pupil Diameter Change

The average pupil diameter for both eyes was calculated in Pupil Cloud and checked every 5 ms in Excel (Microsoft Corporation, Redmond, Washington). Values corresponding to blinks were replaced with 0.0 mm. These data were then imported into CAPTIV‐L2100 (TEA—Tech Ergo Appliquées, Vandoeuvre‐lès‐Nancy, France) along with the scene‐camera videos that contained the corresponding gaze data. The researcher reviewed the scene‐camera footage containing the gaze data and manually categorized each gaze point into specific areas of interest.

To synchronize the time‐series gaze and pupil diameter data, the timestamps of the two datasets were checked; if any discrepancies were found, the timestamps were adjusted to align the starting points. Markers were placed at the start, the 60‐s midpoint, and the end of the simulation task. The gaze and pupil diameter data were exported as time‐series data at 1 Hz. Data collected during blinks were excluded at this stage.

To calculate changes in pupil diameter, a subtraction method was adopted. This method is less susceptible to the influence of baseline correction when dealing with blinks, data loss, or unrealistically small pupil sizes (Mathôt et al. 2018). Because attention control was expected to be unstable immediately before and after the start of the task, the baseline value was defined as the average pupil diameter during the first 50 ms after the start of the task (Mathôt and Vilotijević 2023), and the change in pupil diameter was calculated by subtracting this baseline value from each measured value.

First, the change in pupil diameter when gazing at each area of interest was compared between the two groups. As the analyses in this study were exploratory, formal adjustments for multiple comparisons were not performed. The items included the face, chest, abdomen, right upper limb, left upper limb, right lower limb, left lower limb, bedside monitor, oxygen flowmeter, including suction and air pipes, bedside remote, IV bag including clamp, IV insertion site, and patient information sheet. A mixed‐effects model was used to examine whether changes in pupil diameter differed between the two groups over time. Additionally, to replicate the situation immediately after an emergency admission, the bedside monitor alarms were intentionally left unadjusted. Therefore, it was assumed that some participants might not notice changes in the patient's condition at the 60‐s mark. To address this, participants who gazed at the bedside monitor after 60 s were identified, and a subgroup analysis was conducted.

### 2.8 Statistical Analysis

Statistical comparisons of the demographic characteristics and pupil diameter changes were conducted using JMP 18.2.2 (JMP Statistical Discovery LLC, Cary, North Carolina). Fisher's exact test was used for nominal‐scale demographic variables.

For comparisons of the interval‐scale demographic data and between‐group comparisons of pupil diameter changes, normality was first assessed using the Shapiro–Wilk test. A standard *t*‐test, Welch's *t*‐test, or the Wilcoxon rank‐sum test was applied based on the results. We used a parametric mixed‐effects model because pupil diameter change, the primary outcome and dependent variable, had a hierarchical structure. The fixed effects included group, sex, age, a pre‐event time component (time_pre) capturing the linear trend before 60 s, a post‐event time component (time_post) capturing the linear trend after 60 s, and a post‐event indicator (0 for 0–60 s; 1 for ≥ 61 s). Only interactions between the group and the three time‐related variables were included (i.e., group × time_pre, group × time_post, and group × post‐event indicator). Random intercepts were specified for participant ID, and within‐subject residuals were modeled with an AR(1) correlation structure (Subject = ID; repeated order = time in seconds) to account for between‐subject heterogeneity and within‐subject serial correlation. Model diagnostics included residual‐vs.‐fitted plots, residual‐vs.‐time plots by subject, Q–Q plots, and the empirical variogram.

A significance level of 0.05 was used.

### 2.9 Ethical Statement

This study was conducted in accordance with the Declaration of Helsinki and approved by the Research Ethics Committee of the Kitasato University, School of Nursing (approval no. 2023‐7‐2).

## 3 Results

### 3.1 Participant Demographics

A total of 33 participants were included in the study, with 16 in the skilled nurse group and 17 in the novice nurse group. As the assumption of normality was not met for the variables used in the two‐group comparisons, nonparametric tests were applied for those analyses.

The median age (25th–75th percentile) of the skilled nurse group was 37.5 years (33.0 to 42.0), significantly higher than the novice nurse group's median of 24.0 years (23.0 to 24.0) (*p* < 0.001, effect size *r* = 0.85). Regarding sex, 11 participants (69%) in the skilled nurse group were male, and five (31%) were female, whereas in the novice nurse group, three (18%) were male and 14 (82%) were female, with a significant difference between the groups (*p* < 0.01, *φ* = 0.52). No significant differences were observed between the two groups in terms of the presence or type of vision correction (Table 3).

**TABLE 3:** Participant characteristics.

		Skilled nurses (*n* = 16)		Novice nurses (*n* = 17)		*p*	Effect size
		*n*	%	*n*	%		
Sex	Male	11	69%	3	18%	< 0.01	0.52
	Female	5	31%	14	82%		
Use of vision correction	Yes	12	75%	10	59%	0.465	0.17
	No	4	25%	7	41%		
Type of vision correction	Soft contact lenses	7	58%	8	80%	0.646	0.22
	Glasses	4	33%	2	20%		
	LASIK surgery	1	8%	0	0%		

*Note:* LASIK Surgery stands for Laser‐Assisted In Situ Keratomileusis. Categorical data were analyzed using Fisher's exact test. Percentages may not sum to 100% because of rounding. Statistical significance was defined as *p* < 0.05. Effect sizes are reported as *φ* for 2 × 2 comparisons and Cramér's *V* for larger tables.

### 3.2 Between‐Group Comparison of Pupil Diameter Change

**FIGURE 4:**
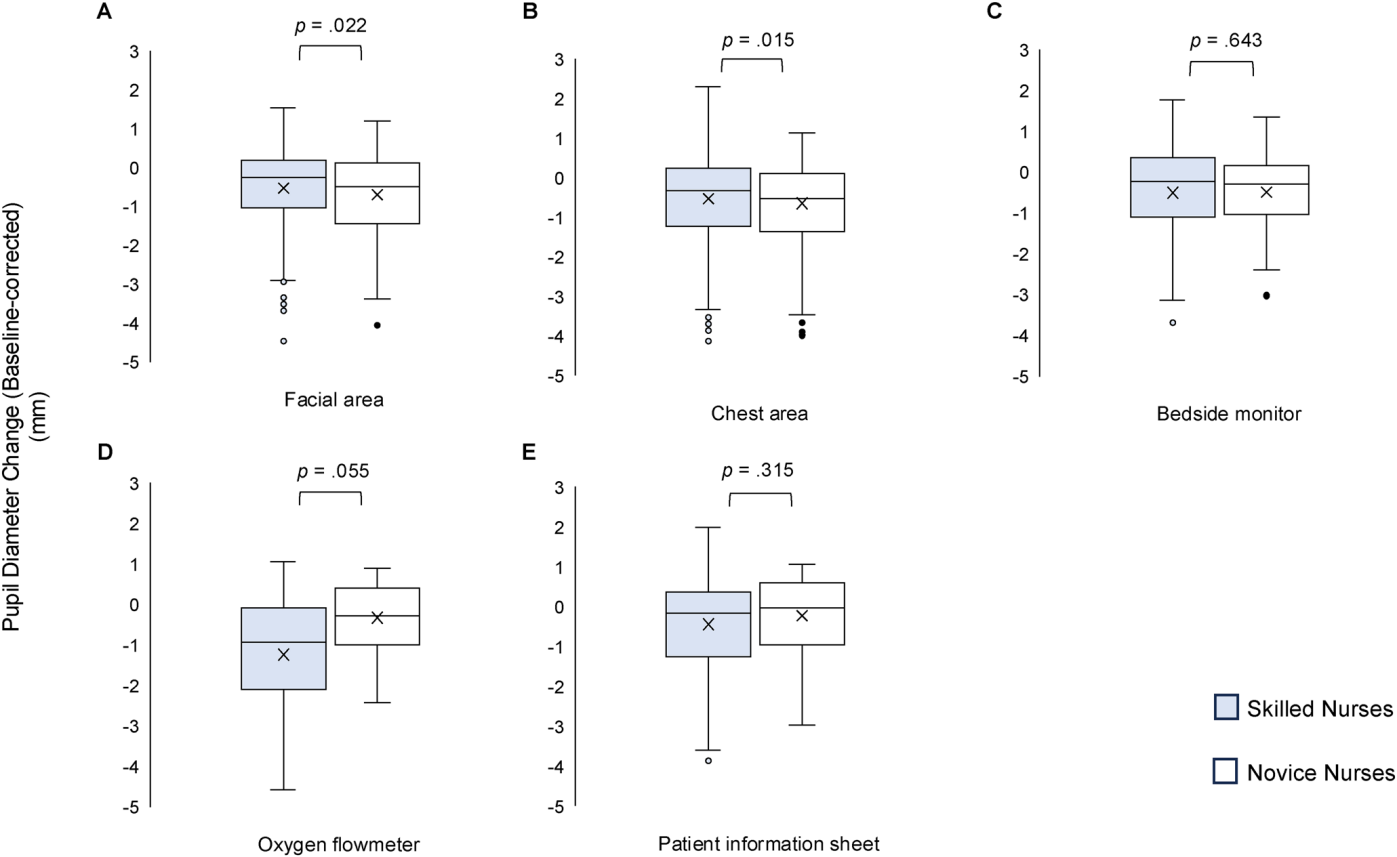
Comparison of pupil diameter changes between the two groups across gaze areas of interest. Panels A–E represent the facial area (A), chest area (B), bedside monitor (C), oxygen flowmeter (D), and patient information sheet (E), respectively. Boxes indicate the interquartile range (IQR; Q1–Q3), and the central horizontal line represents the median. Whiskers extend from Q1 and Q3 to the minimum and maximum observations that fall within 1.5 × IQR; values beyond this range are plotted as outliers (dots). “×” markers denote the mean; the mean line is not shown. Quartiles were computed using Excel's “exclusive median” method. The Wilcoxon rank‐sum test was used for comparisons between the skilled and novice groups. Statistical significance was defined as *p* < 0.05.

Figure 4 compares the pupil diameter changes between the two groups for key gaze items.

When gazing at the facial area, the skilled nurse group showed a significantly smaller change in pupil diameter, with a median of −0.264 mm (25th–75th percentile: −1.041 to −0.208) compared with the novice nurse group, which had a median of −0.491 mm (−1.441 to −0.109) (*p* = 0.022, *r* = 0.40).

Similarly, when gazing at the chest area, the skilled nurse group exhibited a significantly smaller change in pupil diameter, with a median of −0.308 mm (−1.220 to −0.263), compared with the novice nurse group, which had a median of −0.523 mm (−1.339 to −0.104) (*p* = 0.015, *r* = 0.42).

For the other gaze items, no statistically significant differences were observed between the two groups.

### 3.3 Mixed‐Effects Model Analysis of Pupil Diameter Changes During Simulation Task

Overall, residual‐vs.‐fitted/time plots and Q–Q plots revealed no concerning departures, and the empirical variogram supported the AR(1) residual specification.

In the primary mixed‐effects analysis, a significant effect of time_post was observed (*β* = −0.006, SE = 0.002, 95% CI [−0.010, −0.002], *p* = 0.002). This finding indicated that novice nurses showed a decreasing trend in pupil diameter change during the latter half of the observation period. A significant interaction between group (skilled vs. novice) and time_post was also found (*β* = 0.007, SE = 0.003, 95% CI [0.002, 0.013], *p* = 0.010). This interaction indicated that skilled nurses showed slightly larger pupil diameter changes during the latter half of the observation period than novice nurses. In addition, the interaction between the group and the post‐event indicator was significant (*β* = 0.265, SE = 0.134, 95% CI [0.002, 0.529], *p* = 0.048). At the 60‐s transition, skilled nurses showed a larger change in pupil diameter compared with novice nurses. A significant variance was observed for the random effect of participant ID (variance = 0.338, SE = 0.091, 95% CI [0.159, 0.518], *p* < 0.001). The model fit indices were as follows: −2 log‐likelihood (−2LL) = 10051.0, AIC_c_ = 10077.0, and BIC = 10158.8 (Table 4). In the subgroup analysis of participants who gazed at the bedside monitor after 60 s, significant effects were observed for time_post and the group (skilled vs. novice) × time_post interaction, consistent with the primary analysis. The random effect was also significant. In contrast, the group × post‐event indicator interaction was not significant (Table 5). This analysis included 10 skilled nurses and seven novice nurses.

**TABLE 4:** Mixed‐effects model predicting change in pupil diameter.

Effect	Estimate (*β*)	SE	95% CI [LL, UL]	*p*
Fixed effects				
Group (skilled vs. novice)	0.302	0.527	[−0.774, 1.377]	0.571
Sex (female vs. male)	0.363	0.291	[−0.232, 0.957]	0.222
Age (years)	−0.013	0.026	[−0.067, 0.041]	0.615
time_pre	0.003	0.002	[−0.001, 0.007]	0.120
time_post	−0.006	0.002	[−0.010, −0.002]	0.002
post‐event indicator	−0.069	0.094	[−0.253, 0.114]	0.460
Group × time_pre	−0.001	0.003	[−0.006, 0.005]	0.780
Group × time_post	0.007	0.003	[0.002, 0.013]	0.010
Group × post‐event indicator	0.265	0.134	[0.002, 0.529]	0.048
Random effects				
Participant ID	0.338	0.091	[0.159, 0.518]	< 0.001

*Note:* Model fit: −2 Log Likelihood = 10051.0, AIC_c_ = 10077.0, BIC = 10158.8. Unstandardized coefficients are reported. 95% confidence intervals (CIs) are reported. The time variable was decomposed into linear components before and after the 60‐s mark: time_pre represents the linear trend during the pre‐event period (0–60 s), time_post represents the linear trend during the post‐event period (≥ 61 s), and the post‐event indicator variable takes the value 0 for 0–60 s and 1 for ≥ 61 s. Categorical predictors were dummy‐coded with novice (group) and male (sex) as reference levels. Skilled refers to skilled nurses; novice refers to novice nurses. Random effect is reported as variance (not coefficient). For the AR(1) residual structure, the lag‐1 correlation was *ρ̂* = 0.228 (SE = 0.016, 95% CI [0.197, 0.259]), and the residual variance was *σ* ^2^ = 0.745 (SE = 0.018, 95% CI [0.711, 0.781]). Estimation method: REML; software: JMP 18.2.2. Statistical significance was defined as *p* < 0.05.

**TABLE 5:** Mixed‐effects model predicting change in pupil diameter (Subgroup: Participants who gazed at the bedside monitor after 60 s).

Effect	Estimate (*β*)	SE	95% CI [LL, UL]	*p*
Fixed effects				
Group (skilled vs. novice)	0.658	0.662	[−0.765, 2.082]	0.337
Sex (female vs. male)	0.445	0.434	[−0.493, 1.382]	0.324
Age (years)	−0.023	0.032	[−0.091, 0.045]	0.479
time_pre	0.003	0.003	[−0.003, 0.009]	0.355
time_post	−0.007	0.003	[−0.013, 0.000]	0.037
post‐event indicator	−0.090	0.158	[−0.401, 0.221]	0.570
Group × time_pre	0.002	0.004	[−0.006, 0.010]	0.679
Group × time_post	0.009	0.004	[0.000, 0.017]	0.043
Group × post‐event indicator	0.329	0.206	[−0.077, 0.734]	0.112
Random effects				
Participant ID	0.305	0.124	[0.062, 0.548]	0.014

*Note:* Model fit: −2 Log Likelihood = 5345.0, AIC_c_ = 5371.2, BIC = 5444.2. Unstandardized coefficients are reported. 95% confidence intervals (CIs) are reported. The time variable was decomposed into linear components before and after the 60‐s mark: time_pre represents the linear trend during the pre‐event period (0–60 s), time_post represents the linear trend during the post‐event period (≥ 61 s), and the post‐event indicator variable takes the value 0 for 0–60 s and 1 for ≥ 61 s. Categorical predictors were dummy‐coded with novice (group) and male (sex) as reference levels. Skilled refers to skilled nurses; novice refers to novice nurses. Random effect is reported as variance (not coefficient). For the AR(1) residual structure, the lag‐1 correlation was *ρ̂* = 0.263 (SE = 0.022, 95% CI [0.221, 0.306]), and the residual variance was *σ* ^2^ = 0.826 (SE = 0.028, 95% CI [0.774, 0.884]). Estimation method: REML; software: JMP 18.2.2. Subgroup analysis was conducted for participants who gazed at the bedside monitor after 60 s (10 skilled nurses, 7 novice nurses). Statistical significance was defined as *p* < 0.05.

## 4 Discussion

In this study, pupil diameter values were recorded to investigate the characteristics of attention in skilled and novice nurses during their initial observation of a patient urgently admitted with suspected community‐acquired pneumonia.

Between‐group comparisons of pupil diameter change across predefined gaze items revealed that the skilled nurse group showed smaller changes in pupil diameter when gazing at the face and chest, which were defined as key gaze items in this study. The median values in both groups were negative. Pupil diameter changes were calculated by subtracting the baseline—defined as the average pupil diameter during the initial 50 ms after the start of the task—from the value obtained when gazing at each item; therefore, a negative value does not necessarily indicate actual pupil constriction but simply that the measured diameter was smaller than the baseline. These negative values may reflect the use of a baseline pupil diameter measured immediately after task onset, when participants' attention was likely heightened, and the baseline may therefore have been relatively large. Notably, the novice nurse group showed more negative values than the skilled nurse group. This study defined focused attention as a state in which the pupil was dilated. Therefore, although these results do not allow us to conclude definitively that the participants were directing attention to the face and chest, the between‐group comparison suggests that skilled nurses may have allocated attention more intensively to these areas than novice nurses. However, as described in the Methods section, the analyses in this study were exploratory, and no formal adjustments for multiple comparisons were performed; therefore, an increased risk of Type I error should be considered.

Patients with community‐acquired pneumonia are often hospitalized because of symptoms such as cough and dyspnea (Vaughn et al. 2024). Visual inspection of the chest is also considered important by physicians (Shellenberger et al. 2017), and the fact that skilled nurses directed their attention to the face and chest during observation suggests that they may have been assessing the severity of the pneumonia‐related symptoms. In the care of older adults, nurses gaze at patients' faces as part of nonverbal communication (Caris‐Verhallen et al. 1999). Furthermore, because human emotions are often expressed through facial expressions (Ekman et al. 1987), it is possible that skilled nurses not only evaluate the patient's condition but also attempt to engage in emotional communication. Taken together, these findings suggest that, in the absence of alarm sounds, nurses may prioritize the patient's face and chest over the bedside monitor.

Focused attention to the face and chest, as suggested by the findings in skilled nurses, is an essential nursing observation skill for forming a first impression of a severely ill patient.

To investigate whether the characteristics of each group were associated with pupil diameter changes, a mixed‐effects model analysis was conducted. As a significant variance was observed for the random effect of participant ID, the data on pupil diameter changes appear to have involved substantial inter‐individual variability. In mixed‐effects models, such individual differences are statistically adjusted for; therefore, the resulting fixed effects can be interpreted as estimates of population‐level means after controlling for these differences. For time_post, novice nurses exhibited a tendency for their pupil diameter changes to decrease over time. In addition, the interaction between group and time_post indicated that skilled nurses showed a slightly more positive trend in pupil diameter change after 60 s than novice nurses. In the simulation task used in this study, the bedside monitor parameters were intentionally set to deteriorate at 60 s, and the respiratory sound data were modified to reflect an increased respiratory rate. Although it cannot be determined whether all skilled nurses recognized these changes, they may have intensified their observational efforts in response to perceived alterations in the patient's condition. The significant interaction between the group and the post‐event indicator further suggests that skilled nurses who detected the 60‐s change may have responded more strongly than novice nurses. Given that the skilled nurses had completed training in clinical reasoning and physical assessment, it is conceivable that they were selectively directing attention and conducting a comprehensive evaluation of the patient's overall condition. However, the present study did not sufficiently examine the specific characteristics of the skilled nurse group that contributed to pupil diameter changes, and this remains an important subject for future investigation.

This study simulated an initial observation scenario in an emergency admission before alarms had been set. Some participants may not have noticed changes in the bedside monitor until after 60 s; therefore, a subgroup analysis was conducted for those who gazed at the monitor after 60 s. The results showed largely similar trends to those in the overall analysis, suggesting that this factor was unlikely to have substantially influenced the main findings. The absence of a significant interaction between group and the post‐event indicator may be attributable to the smaller between‐group differences in this subgroup, which consisted of participants who may have noticed the change. However, because the subgroup had a small sample size and wider confidence intervals, the precision of the estimates may have been lower than in the overall analysis.

In nursing, a previous study adopted pupil diameter change as an indicator of cognitive load (Cabrera‐Mino et al. 2019). In that study, during a 10‐min observation, pupil diameter changes were compared between nursing students nearing graduation and nurses with over 5 years of clinical experience working in ICUs or emergency departments as they performed tasks such as oxygen administration to patients with heart failure. Nursing students exhibited greater pupil diameter changes during tasks such as adjusting the bed position, administering oxygen, connecting the oxygen tube to the flowmeter, and auscultating lung sounds. Although the present findings may appear to contradict those of the previous study, this discrepancy is probably attributable to differences in the simulated scenarios and observation duration. In the present study, we emphasized the early recognition of patient deterioration; therefore, the scenario simulated an emergency admission and used a 120‐s observation period instead of 10 min. In addition, the scenario was designed so that the patient's condition deteriorated during the task. Accordingly, the task may have required meticulous attention. By contrast, during longer observation periods, factors such as anxiety and stress may exert a stronger influence on pupil diameter than attentional processes alone. Researchers using pupil diameter change as an outcome measure should consider the attentional mechanisms of interest and carefully define the task stimuli and participant attributes. The previous study focused on patients with heart failure, whereas the present study examined patients with pneumonia. Differences in patients' conditions, symptoms, and complaints may influence pupil diameter changes. Therefore, future research should examine pupil diameter changes in relation to these factors, along with other physiological indicators, to enable a more comprehensive assessment, particularly because such changes may reflect physiological processes other than attention.

Because observation techniques are inherently difficult to verbalize, VR‐based training materials may be an effective means of passing these skills on to novice nurses. For example, a study comparing visually enhanced and standard VR materials for anatomy learning found that the visually enhanced materials improved visual search performance (Bautista et al. 2025). The present findings not only highlight the observational abilities of skilled nurses but also provide foundational data for designing VR materials that emphasize specific areas requiring focused attention. These findings may be particularly useful for teaching novice nurses in the systematic observation of patients with deteriorating conditions. However, because this study focused on the initial 120 s of observation and used a nonfunctional manikin, it is difficult to generalize the findings beyond the initial assessment of patients with community‐acquired pneumonia. Further research involving high‐fidelity simulators, actual patients, and scenarios with different pathological conditions will be essential to enhance the clinical generalizability of these findings.

### 4.1 Limitations of the Study

Because of the experimental design, we did not control for sex and age. A previous study reported no significant sex differences in pupil diameter (Jones 1990). In addition, neither sex nor age showed statistically significant fixed effects in the mixed‐effects model used in this study. However, because the distributions of sex and age differed significantly between the two groups, the potential effects of these variables may not have been fully accounted for. Therefore, future research should consider increasing the sample size and adopting study designs that control for sex and age.

In line with previous research, the baseline value was set based on measurements taken shortly after the beginning of the task. However, if a resting period had been provided before the task and used as the baseline, the results could have been clearer.

This study used a human‐shaped manikin as the patient, which limited the range of possible responses, including changes in facial expressions and breathing patterns. Real patients exhibit diverse reactions, and nurses' attentional processes may vary accordingly. Additionally, although the values displayed on the bedside monitor worsened at the 60‐s mark, no alarms were set in order to replicate the conditions immediately after hospital admission. Consequently, some participants may not have noticed the changes in the patient's condition shown on the monitor. Because this study focused on nurses' observation techniques, subsequent actions in response to their observations were not investigated. Attentional processes may differ when participants are required not only to observe but also to act based on their observations. Therefore, future research should investigate both observation and subsequent clinical actions using high‐fidelity simulators or actual patients. We prioritized the early detection of patient abnormalities and therefore selected an observation time of 120 s. However, in actual clinical settings, observation continues over longer periods. The results of this study could serve as foundational data for future studies involving extended observation or for the development of VR materials to train nurses in managing sudden changes in patient conditions.

## 5 Conclusions

During the initial assessment of a patient urgently admitted with suspected community‐acquired pneumonia, both skilled and novice nurses showed negative pupil diameter changes when gazing at the face and chest, with the skilled nurse group showing smaller negative changes. Moreover, the mixed‐effects model showed that skilled nurses exhibited slightly larger pupil diameter changes in the later phase of the task compared with novice nurses. Future research should clarify the evolution of attentional states in more realistic clinical environments.

## Author Contributions

**Shoya Kamijo:** conceptualization, data curation, formal analysis, funding acquisition, project administration, writing – original draft, writing – review and editing. **Shinji Matsutani:** conceptualization, formal analysis, supervision, writing – review and editing. **Eijun Nakayama:** conceptualization, supervision, writing – review and editing. **Naoya Ohtani:** conceptualization, interpretation of results, writing – review and editing. All authors have read and approved the final manuscript.

## Funding

This work was supported by JSPS KAKENHI (JP23K09890).

## Ethics Statement

This study was conducted in accordance with the Declaration of Helsinki and approved by the Research Ethics Committee of the Kitasato University, School of Nursing (Approval No. 2023‐7‐2, date of approval: September 29, 2023).

## Conflicts of Interest

The authors declare no conflicts of interest.

## Data Availability

The data that support the findings of this study are available on request from the corresponding author. The data are not publicly available due to privacy or ethical restrictions.

## References

[jjns70076-bib-0001] Alastalo, M. , L. Salminen , R. L. Lakanmaa , and H. Leino‐Kilpi . 2017. “Seeing Beyond Monitors‐Critical Care Nurses' Multiple Skills in Patient Observation: Descriptive Qualitative Study.” Intensive & Critical Care Nursing 42: 80–87. 10.1016/j.iccn.2017.03.004. 28363593

[jjns70076-bib-0002] Alnæs, D. , M. H. Sneve , T. Espeseth , T. Endestad , S. H. P. van de Pavert , and B. Laeng . 2014. “Pupil Size Signals Mental Effort Deployed During Multiple Object Tracking and Predicts Brain Activity in the Dorsal Attention Network and the Locus Coeruleus.” Journal of Vision 14, no. 4: 1. 10.1167/14.4.1. 24692319

[jjns70076-bib-0003] Atkinson, R. C. , and R. M. Shiffrin . 1968. “Human Memory: A Proposed System and Its Control Processes.” In The Psychology of Learning and Motivation: Advances in Research and Theory, edited by K. W. Spence and J. T. Spence , vol. 2, 89–195. Academic Press. 10.1016/s0079-7421(08)60422-3.

[jjns70076-bib-0004] Bari, A. , S. Xu , M. Pignatelli , et al. 2020. “Differential Attentional Control Mechanisms by Two Distinct Noradrenergic Coeruleo‐Frontal Cortical Pathways.” Proceedings of the National Academy of Sciences of the United States of America 117, no. 46: 29080–29089. 10.1073/pnas.2015635117. 33139568 PMC7682591

[jjns70076-bib-0005] Bautista, L. E. , F. Maradei , P. A. G. Vargas , and L. M. Moreno . 2025. “Use of Color in Visual Coding of Information for Augmented Reality Environments.” Virtual Reality 29, no. 2: 67. 10.1007/s10055-025-01132-1.

[jjns70076-bib-0006] Bradley, M. M. , L. Miccoli , M. A. Escrig , and P. J. Lang . 2008. “The Pupil as a Measure of Emotional Arousal and Autonomic Activation.” Psychophysiology 45, no. 4: 602–607. 10.1111/j.1469-8986.2008.00654.x. 18282202 PMC3612940

[jjns70076-bib-0007] Cabrera‐Mino, C. , M. A. Shinnick , and S. Moye . 2019. “Task‐Evoked Pupillary Responses in Nursing Simulation as an Indicator of Stress and Cognitive Load.” Clinical Simulation in Nursing 31: 21–27. 10.1016/j.ecns.2019.03.009.

[jjns70076-bib-0008] Caris‐Verhallen, W. M. , A. Kerkstra , and J. M. Bensing . 1999. “Non‐Verbal Behaviour in Nurse‐Elderly Patient Communication.” Journal of Advanced Nursing 29, no. 4: 808–818. 10.1046/j.1365-2648.1999.00965.x. 10215971

[jjns70076-bib-0009] Cioffi, J. 2001. “Clinical Simulations: Development and Validation.” Nurse Education Today 21, no. 6: 477–486. 10.1054/nedt.2001.0584. 11466011

[jjns70076-bib-0010] Corbetta, M. , E. Akbudak , T. E. Conturo , et al. 1998. “A Common Network of Functional Areas for Attention and Eye Movements.” Neuron 21, no. 4: 761–773. 10.1016/s0896-6273(00)80593-0. 9808463

[jjns70076-bib-0011] Corbetta, M. , and G. L. Shulman . 2002. “Control of Goal‐Directed and Stimulus‐Driven Attention in the Brain.” Nature Reviews. Neuroscience 3, no. 3: 201–215. 10.1038/nrn755. 11994752

[jjns70076-bib-0012] Drew, T. , M. L. H. Võ , and J. M. Wolfe . 2013. “The Invisible Gorilla Strikes Again: Sustained Inattentional Blindness in Expert Observers.” Psychological Science 24, no. 9: 1848–1853. 10.1177/0956797613479386. 23863753 PMC3964612

[jjns70076-bib-0013] Ekman, P. , W. V. Friesen , M. O'Sullivan , et al. 1987. “Universals and Cultural Differences in the Judgments of Facial Expressions of Emotion.” Journal of Personality and Social Psychology 53, no. 4: 712–717. 10.1037//0022-3514.53.4.712. 3681648

[jjns70076-bib-0014] Faul, F. , E. Erdfelder , A. G. Lang , and A. Buchner . 2007. “G*Power 3: A Flexible Statistical Power Analysis Program for the Social, Behavioral, and Biomedical Sciences.” Behavior Research Methods 39, no. 2: 175–191. 10.3758/bf03193146. 17695343

[jjns70076-bib-0015] Ghosh, S. , and J. H. R. Maunsell . 2024. “Locus Coeruleus Norepinephrine Contributes to Visual‐Spatial Attention by Selectively Enhancing Perceptual Sensitivity.” Neuron 112, no. 13: 2231–2240.e5. 10.1016/j.neuron.2024.04.001. 38701788 PMC11223979

[jjns70076-bib-0016] Grundgeiger, T. , P. Sanderson , H. G. MacDougall , and B. Venkatesh . 2010. “Interruption Management in the Intensive Care Unit: Predicting Resumption Times and Assessing Distributed Support.” Journal of Experimental Psychology. Applied 16, no. 4: 317–334. 10.1037/a0021912. 21198250

[jjns70076-bib-0017] Hamilton, B. E. , and E. Manias . 2007. “Rethinking Nurses' Observations: Psychiatric Nursing Skills and Invisibility in an Acute Inpatient Setting.” Social Science and Medicine 65, no. 2: 331–343. 10.1016/j.socscimed.2007.03.025. 17459545

[jjns70076-bib-0018] He, Z. , J. L. Marquard , and P. L. Henneman . 2014. “How Do Interruptions Impact Nurses' Visual Scanning Patterns When Using Barcode Medication Administration Systems?” AMIA Annual Symposium Proceedings 2014: 1768–1776. 25954449 PMC4419886

[jjns70076-bib-0019] Henneman, E. A. , A. Gawlinski , C. Nicholas , K. McAfee , J. L. Marquard , and C. Andrzejewski . 2017. “Identification of Transfusion‐Associated Circulatory Overload: An Eye‐Tracking Study.” Clinical Simulation in Nursing 13, no. 12: 675–679. 10.1016/j.ecns.2017.05.004.

[jjns70076-bib-0020] Hofmaenner, D. A. , A. Herling , S. Klinzing , et al. 2021. “Use of Eye Tracking in Analyzing Distribution of Visual Attention Among Critical Care Nurses in Daily Professional Life: An Observational Study.” Journal of Clinical Monitoring and Computing 35, no. 6: 1511–1518. 10.1007/s10877-020-00628-2. 33296061 PMC7724778

[jjns70076-bib-0021] Jones, A. , and M.‐J. Johnstone . 2017. “Inattentional Blindness and Failures to Rescue the Deteriorating Patient in Critical Care, Emergency and Perioperative Settings: Four Case Scenarios.” Australian Critical Care 30, no. 4: 219–223. 10.1016/j.aucc.2016.09.005. 27720335

[jjns70076-bib-0022] Jones, R. 1990. “Do Women and Myopes Have Larger Pupils?” Investigative Ophthalmology & Visual Science 31, no. 7: 1413–1415. 2365574

[jjns70076-bib-0023] Joshi, S. , and J. I. Gold . 2020. “Pupil Size as a Window on Neural Substrates of Cognition.” Trends in Cognitive Sciences 24, no. 6: 466–480. 10.1016/j.tics.2020.03.005. 32331857 PMC7271902

[jjns70076-bib-0024] Joshi, S. , Y. Li , R. M. Kalwani , and J. I. Gold . 2016. “Relationships Between Pupil Diameter and Neuronal Activity in the Locus Coeruleus, Colliculi, and Cingulate Cortex.” Neuron 89, no. 1: 221–234. 10.1016/j.neuron.2015.11.028. 26711118 PMC4707070

[jjns70076-bib-0025] Kamijo, S. , E. Nakayama , M. Kobayashi , and S. Matsutani . 2023. “A Scoping Review of the Gaze of Nurses With Proficient Skills in Patient Observation.” Japanese Journal of Nursing Art and Science 22: 51–62. (in Japanese). 10.18892/jsnas.22.0_51.

[jjns70076-bib-0026] Koh, R. Y. I. , T. Park , C. D. Wickens , L. T. Ong , and S. N. Chia . 2011. “Differences in Attentional Strategies by Novice and Experienced Operating Theatre Scrub Nurses.” Journal of Experimental Psychology. Applied 17, no. 3: 233–246. 10.1037/a0025171. 21942313

[jjns70076-bib-0027] Liaw, S. Y. , A. Scherpbier , P. Klainin‐Yobas , and J. J. Rethans . 2011. “A Review of Educational Strategies to Improve Nurses' Roles in Recognizing and Responding to Deteriorating Patients.” International Nursing Review 58, no. 3: 296–303. 10.1111/j.1466-7657.2011.00915.x. 21848774

[jjns70076-bib-0028] Liu, K. , W. Zhang , W. Li , T. Wang , and Y. Zheng . 2023. “Effectiveness of Virtual Reality in Nursing Education: A Systematic Review and Meta‐Analysis.” BMC Medical Education 23, no. 1: 710. 10.1186/s12909-023-04662-x. 37770884 PMC10540340

[jjns70076-bib-0029] Lynn, L. A. , and J. P. Curry . 2011. “Patterns of Unexpected In‐Hospital Deaths: A Root Cause Analysis.” Patient Safety in Surgery 5, no. 1: 3. 10.1186/1754-9493-5-3. 21314935 PMC3045877

[jjns70076-bib-0030] Lynn, M. R. 1986. “Determination and Quantification of Content Validity.” Nursing Research 35, no. 6: 382–385. 10.1097/00006199-198611000-00017. 3640358

[jjns70076-bib-0031] Marquard, J. L. , P. L. Henneman , Z. He , J. Jo , D. L. Fisher , and E. A. Henneman . 2011. “Nurses' Behaviors and Visual Scanning Patterns May Reduce Patient Identification Errors.” Journal of Experimental Psychology. Applied 17, no. 3: 247–256. 10.1037/a0025261. 21942314

[jjns70076-bib-0032] Marquard, J. L. , J. Jo , P. L. Henneman , D. L. Fisher , and E. A. Henneman . 2013. “Can Visualizations Complement Quantitative Process Analysis Measures? A Case Study of Nurses Identifying Patients Before Administering Medications.” Journal of Cognitive Engineering and Decision Making 7, no. 2: 198–210. 10.1177/1555343412457683.

[jjns70076-bib-0033] Mathôt, S. , J. Fabius , E. Van Heusden , and S. Van der Stigchel . 2018. “Safe and Sensible Preprocessing and Baseline Correction of Pupil‐Size Data.” Behavior Research Methods 50, no. 1: 94–106. 10.3758/s13428-017-1007-2. 29330763 PMC5809553

[jjns70076-bib-0034] Mathôt, S. , and A. Vilotijević . 2023. “Methods in Cognitive Pupillometry: Design, Preprocessing, and Statistical Analysis.” Behavior Research Methods 55, no. 6: 3055–3077. 10.3758/s13428-022-01957-7. 36028608 PMC10556184

[jjns70076-bib-0035] Ministry of Health, Labour and Welfare . n.d. “Medical Care.” https://www.mhlw.go.jp/english/policy/health‐medical/medical‐care/index.html.

[jjns70076-bib-0036] Miyazaki, T. , K. Hirano , K. Ichihara , et al. 2023. “Community‐Acquired Pneumonia Incidence in Adults Aged 18 Years and Older in Goto City, Japan: A Prospective Population‐Based Study.” Chest Pulmonary 1, no. 2: 100007. 10.1016/j.chpulm.2023.100007. 42548386 PMC13418085

[jjns70076-bib-0037] Murphy, P. R. , R. G. O'Connell , M. O'Sullivan , I. H. Robertson , and J. H. Balsters . 2014. “Pupil Diameter Covaries With BOLD Activity in Human Locus Coeruleus.” Human Brain Mapping 35, no. 8: 4140–4154. 10.1002/hbm.22466. 24510607 PMC6869043

[jjns70076-bib-0038] Okane, R. , T. Hasegawa , M. Ohira , M. Komatsu , S. Saito , and K. Yokoyama . 2022. “Characteristics of Observations by Expert Nurses of Mechanically Ventilated Patients.” Japanese Journal of Ergonomics 58, no. 4: 186–194. (in Japanese). 10.5100/jje.58.186.

[jjns70076-bib-0039] Privitera, M. , K. D. Ferrari , L. M. von Ziegler , et al. 2020. “A Complete Pupillometry Toolbox for Real‐Time Monitoring of Locus Coeruleus Activity in Rodents.” Nature Protocols 15, no. 8: 2301–2320. 10.1038/s41596-020-0324-6. 32632319

[jjns70076-bib-0040] Schein, R. M. , N. Hazday , M. Pena , B. H. Ruben , and C. L. Sprung . 1990. “Clinical Antecedents to In‐Hospital Cardiopulmonary Arrest.” Chest 98, no. 6: 1388–1392. 10.1378/chest.98.6.1388. 2245680

[jjns70076-bib-0041] Shellenberger, R. A. , B. Balakrishnan , S. Avula , A. Ebel , and S. Shaik . 2017. “Diagnostic Value of the Physical Examination in Patients With Dyspnea.” Cleveland Clinic Journal of Medicine 84, no. 12: 943–950. 10.3949/ccjm.84a.16127. 29244648

[jjns70076-bib-0042] Strauch, C. , C. A. Wang , W. Einhäuser , S. Van der Stigchel , and M. Naber . 2022. “Pupillometry as an Integrated Readout of Distinct Attentional Networks.” Trends in Neurosciences 45, no. 8: 635–647. 10.1016/j.tins.2022.05.003. 35662511

[jjns70076-bib-0043] Tejada, S. , A. Romero , and J. Rello . 2018. “Community‐Acquired Pneumonia in Adults: What's New Focusing on Epidemiology, Microorganisms and Diagnosis?” Erciyes Tıp Dergisi 40, no. 4: 177–182. 10.5152/etd.2018.18128.

[jjns70076-bib-0044] Vaughn, V. M. , R. P. Dickson , J. K. Horowitz , and S. A. Flanders . 2024. “Community‐Acquired Pneumonia: A Review.” Journal of the American Medical Association 332, no. 15: 1282–1295. 10.1001/jama.2024.14796. 39283629

[jjns70076-bib-0045] Waugh, N. C. , and D. A. Norman . 1965. “Primary Memory.” Psychological Review 72: 89–104. 10.1037/h0021797. 14282677

[jjns70076-bib-0046] Xia, R. , X. Chen , T. A. Engel , and T. Moore . 2024. “Common and Distinct Neural Mechanisms of Attention.” Trends in Cognitive Sciences 28, no. 6: 554–567. 10.1016/j.tics.2024.01.005. 38388258 PMC11153008

